# Comparative Survey of Holding Positions for Reducing Vaccination Pain in Young Infants

**DOI:** 10.1155/2017/3273171

**Published:** 2017-01-26

**Authors:** Hui-Chu Yin, Shao-Wen Cheng, Chun-Yuh Yang, Ya-Wen Chiu, Yi-Hao Weng

**Affiliations:** ^1^Department of Nursing, Chang Gung Memorial Hospital, College of Nursing, Chang Gung University, Taipei 105, Taiwan; ^2^Department of Pediatrics, Chang Gung Memorial Hospital, College of Medicine, Chang Gung University, Taipei 105, Taiwan; ^3^Department of Public Health, Kaohsiung Medical University, Kaohsiung 807, Taiwan; ^4^Master Program in Global Health and Development, College of Public Health, Taipei Medical University, Taipei 11031, Taiwan

## Abstract

*Background*. Infant holding position may reduce vaccination pain. However, the optimal position for young infants remains controversial.* Objectives*. To compare the effectiveness of holding infants in the supine position and the effectiveness of holding infants in upright position for relieving acute pain from vaccine injection.* Methods*. This prospective cohort study enrolled 6–12-week-old healthy infants. We examined infant pain responses by evaluating the following three categories: (1) crying, (2) irritability, and (3) facial expression.* Results*. In total, 282 infants were enrolled, with 103 and 179 held in the supine and upright positions, respectively. At 30 s after vaccination, the infants in the supine position showed a larger decrease in crying (*p* < 0.001), irritability (*p* = 0.002), and pained facial expression (*p* = 0.001) than did those in the upright position. However, there was no significant difference in pain response between two groups at 180 s after intervention.* Conclusion*. In 2-month-old infants, the supine position may reduce acute pain more effectively than does the upright position. Our findings provide a clinical strategy for relieving vaccination pain in young infants.

## 1. Introduction

Young infants require vaccination against several diseases, and such vaccinations are implemented worldwide. The efficiency of vaccines in disease prevention is well documented [1]. However, hesitancy exists among parents because of the adverse effects of vaccination, and research on this topic is increasing [2, 3]. When parents perceive an unfavorable experience during vaccination, they may become reluctant to return for follow-up vaccinations and boosters in a timely manner.

Vaccination is one of the most common painful procedures. Pain during infancy can have long-term effects on physiological and behavioral responses to vaccination [4]. Various interventions—physical, psychological, and pharmacological—have been evaluated for reducing vaccination pain in infants [5–9]. Intake of sweet-tasting solutions, such as sucrose or glucose, is helpful in relieving acute pain from injection [10–13]. In addition, breastfeeding has been shown to have analgesic effects [14, 15]. Although sweet solutions and breastfeeding are well recommended for reducing acute pain from vaccine injection [15], there are limitations of use in clinical practice. Long administration period of sweet solutions makes it more difficult for routine use in a busy setting. Furthermore, breastfeeding is only working on selected conditions, such as cultural acceptance of breastfeeding during immunization. By contrast, physical interventions for pain relief are the least expensive and easiest to incorporate into practice when compared with psychological or pharmacological interventions [16]. When sweet solutions and breastfeeding are not available, physical interventions can serve as an alternative method to reduce acute pain from injection. Swaddling, shushing, swinging, sucking, and positioning are commonly used to reduce acute pain from vaccination [17]. However, these physical interventions for acute pain during vaccination in young infants have been rarely surveyed [17, 18]. Thus far, the effects of physical interventions on responses to vaccination are debated [19].

In clinical practice, because parents are concerned regarding the vaccination pain caused to their infants, they generally hold their infants to comfort them [15, 20]. Holding reduces responses to painful procedures [21–23] and is a means for parents to distract and calm their infants. According to the recommendation of World Health Organization (WHO), infants and young children should be held by caregivers during vaccination [15]. Nevertheless, best position is not well defined yet. For young children, holding with upright position is more effective in reducing acute pain from vaccine injection [24]. However, the position most favorable for young infants is unclear.

To date, an investigation of comparison between holding with supine and holding with upright positions is lacking. Because verifying the most favorable position is necessary for relieving vaccination pain in young infants, this study compared two positions to reduce acute pain in 2-month-old infants. The current results can guide evidence-based interventions to reduce iatrogenic pain caused by vaccination.

## 2. Methods

### 2.1. Study Design

**Figure 1:**
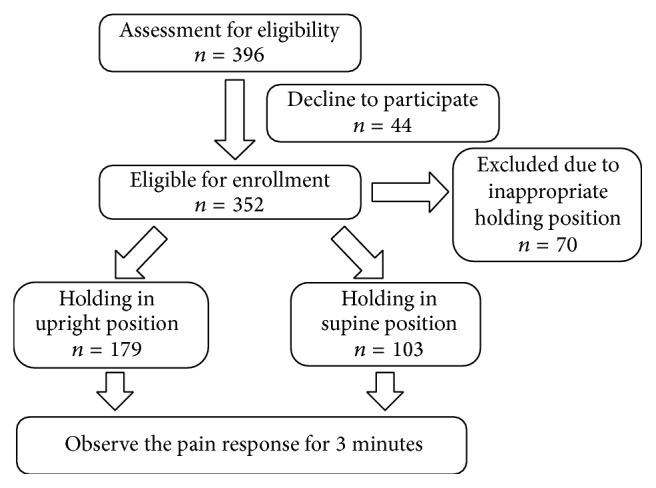
Schematic of study interventions and examinations.

This prospective cohort study involved exploratory research conducted for the intervention of infant holding and examination of infant pain responses after regular vaccination. The flowchart of the study is presented in Figure 1. Healthy infants vaccinated at 6–12 weeks of age were enrolled. Those admitted to the neonatal intensive care and with a gestational age < 34 weeks, birth weight < 2000 g, or illness (such as significant congenital anomaly) were excluded. The study was conducted in the well-baby clinic of Chang Gung Memorial Hospital, Taipei, Taiwan, between July 2014 and April 2015. The institutional review board of Chang Gung Memorial Hospital approved the study protocol. Informed consent was obtained from the parents of the enrolled infants.

### 2.2. Vaccinations

In this study, the injected vaccines were 13-valent pneumococcal conjugate vaccine (PCV13; Prevenar 13™; Pfizer, NY, USA) and DTPa-IPV/Hib (diphtheria toxoid, tetanus toxoid, acellular pertussis, polio, and* Haemophilus influenzae* type b; Pediacel™; Sanofi Pasteur, Lyon, France). The injection procedure, including skin cleaning, injection location, injection pressure, and total injection time, was standardized for all vaccinations to maintain consistency. For skin cleaning, they applied 70% alcohol-based solution on a single-use swab and allowed it to dry completely before injection. The clinical nurses administered DTPa-IPV/Hib in right thighs and then PCV13 in alternate thighs [25]. Each vaccine was administered into the vastus lateralis muscle on the front of the thigh. The injection was rapid. Aspiration was done before pushing the syringe because this is a part of nursing guideline to avoid intravenous injection. Five nurses in charge of injection were trained and accredited by practicing the procedure at least three times before the study commenced. All nurses trained for the study have given injections for at least 3 years of clinical experience in vaccine injection. All five nurses were equally split between groups. After vaccination, manual stimulation of the injection site was not allowed.

### 2.3. Holding Intervention

We studied two positions—supine and upright. Parents selected the holding position and held their infants in that position throughout the intervention period. They were instructed to hold their infants in a manner they were accustomed to and most comfortable with. In general, both groups were in chest-to-chest position. When the parents held the infants in the supine position, they were asked to lay the head against one shoulder for support and to hold the bottom with one hand on the side that their infants were resting on. In addition, the other hand was to be held against the back, which was pressed softly for further support. Regarding infants held in the upright position, their back was against their parents' chest, and they were held upright for 180 s after injection. Inappropriate positions, such as side position or change during the intervention, were excluded. Gentle patting and rocking were allowed while holding. No specific instructions were given regarding how firmly the infants were to be held, whether they were to be cradled, whether they were to be talked to or patted, and whether they sucked on a pacifier. Clinicians were not involved in holding the infants. Infants with a position not qualified with supine or upright position were excluded in the final analysis (Figure 1).

### 2.4. Measures

In this study, we examined infant pain responses by measuring the following three categories: (1) crying, (2) irritability, and (3) facial expression. For each infant, the minimal and maximal grades of each pain category were 0 and 2, respectively. The pain scale used in this study was modified from published assessment tools (Table 1), including the CRIES observational assessment tool and the FLACC measurement tool [4].

A well-trained nurse performed the standardized observational pain scale measurements. This nurse was not involved in vaccine injection and did not realize the study purpose of comparison for holding position. Pain scale measurements were taken at baseline (before injection) and 0 (immediately after injection), 30, and 180 s after vaccination for each infant.

### 2.5. Validity and Reliability

The content of our pain scale was established by three experts. Their expertise included nursing education, vaccination, and clinical nursing. Each expert had more than 20 years of work experience in their field. Furthermore, the validity of our pain scale was examined by three additional experts each with more than 15 years of clinical experience in nursing. They examined whether the contents were suitable and the wordings were appropriate. After adjustments based on the experts' advice, the pain scale was piloted in a group of 30 infants to estimate internal consistency through Cronbach's alpha. The content validity index was 0.97 and Cronbach's alpha was 0.95, indicating sufficient validity.

### 2.6. Statistical Analyses

Statistics were compiled using a commercially available program (SPSS 19.0 for Windows; SPSS Inc., Chicago, IL, USA). Categorical variables were analyzed using the chi-square test. Continuous variables were compared using Student's* t*-test. Significance was defined as* p* < 0.05. A calculated sample size of at least 50 participants in each group was expected to show a 50% difference in pain scores with a power of 80% and alpha of 0.05.

## 3. Results

### 3.1. Demographics

Overall, 396 infants were eligible for enrollment (Figure 1). Among them, 44 declined to participate and 70 were excluded due to inappropriate holding position. In total, 282 infants were enrolled in this study, of whom 103 and 179 were held in the supine and upright positions, respectively (Table 2). Sex, age, person holding the infants, sucking on a soother after injection, and number of vaccinations did not differ significantly between the infants held in both positions. The average age was 67.5 days. Most infants received both vaccines (82.6%), whereas 5.7% and 11.7% received only PCV13 and DTPa-IPV/Hib, respectively.

### 3.2. Pain Scale

Pain scores are compared between the supine and upright positions in Table 3. Scores of facial expression were significantly higher in supine position than in the upright position at both the baseline before injection and the time immediately after injection. By contrast, scores of facial expression were significantly lower in supine position than in the upright position at 30 s after intervention. As for the scores of crying and irritability, there was no significant difference between two groups at both the baseline before injection and the time immediately after injection. However, scores of crying and irritability were significantly lower in supine position than in the upright position at 30 s after intervention. At 180 s after intervention, there was no significant difference in the scores of crying, irritability, and facial expression between two groups.

Table 4 shows the dichotomized pain scores between the supine and upright positions. Scores 1 and 2 were categorized as positive pain responses. By contrast, score 0 was classified as a negative pain response. At 30 s after vaccination, the crying, irritability, and facial expression scores of the infants held in the supine position were significantly lower than those of the infants held in the upright position. At 180 s after vaccination, pain scores did not differ significantly between the two groups.

In addition, there was a significant difference in facial expression between single and multiple injections (data not shown). Infants receiving multiple injections had more pain response in facial expression than those receiving single injection.

### 3.3. Pain Reduction

A composite pain score was defined as a sum of all grades obtained in the following three categories: crying, irritability, and facial expression. Pain reduction was considered to be a decrease in composite pain score compared with that immediately after last injection.  Table 5 presents a comparison of pain reductions by the composite grade. At 30 s after vaccination, pain reduction was more common in infants in supine position than upright position. At 180 s after vaccination, there was no significant difference in pain reduction between supine and upright positions.

## 4. Discussion

Although several distraction stimuli have been reported as reducing acute pain during vaccination, few studies have examined the effect of the position in young infants [26, 27]. To our knowledge, this is the first study comparing the effectiveness of the supine and the effectiveness of upright positions for vaccination pain reduction among infants aged approximately 2 months. The review board of our institution accepted that we enrolled a large number of infants to increase the power of sample size. Our study used crying, irritability, and facial expression as indices of acute pain response because they are major factors that parents are concerned with during vaccination. These factors have been widely recognized as sensitive and specific nonverbal pain indicators [4]. To prevent potential variations in a single index, we analyzed these factors separately in addition to a composite pain scale [28]. In addition, we recruited an independent observer blinded to the study purpose for minimizing possible pain assessment-related bias.

Contact such as holding can reduce response to painful procedures [29–31]. Holding by the caregiver is recommended by the WHO for infants and young children receiving vaccine injection [15]. However, it is not clear which holding position is the best. A systematic review found that the upright position is more effective for reducing acute pain in children during vaccination than the supine position is [26]. However, this effect was not observed in infants aged 2–6 months [27]. By contrast, our data demonstrated that holding in the supine position reduced acute pain more effectively than holding in the upright position did in infants aged 2 months. These differences may be explained as follows. The neck muscles of 2-month-old infants are relatively weak and poorly controlled; infants this young cannot balance their own head efficiently [32], and they require external neck support when being held. Thus, infants at this age may dislike being held in the upright position when experiencing pain from an injection. In the next few months of age, head stability improves steadily, though infants may still require neck support. Around this age, infants gradually gain effective control of their heads, and they may start disliking the supine position. Consequently, the upright position is more effective for reducing pain in older infants and children [24, 33].

Although the upright position did not alleviate pain as effectively as the supine position did in our study, approximately two-thirds of the parents preferred holding their babies in the upright position. Because head stability is underdeveloped during early infancy, we suggest that the upright position is not suitable for reducing pain during vaccination [34].

In this study, the pain response was relatively high. We speculate two possible reasons. First, the procedure of aspiration may cause longer contact time and lateral movement of the needle [5, 15]. Although aspiration is present in the nursing guideline of our institute, it is no longer recommended by the WHO [15]. Second, our study did not conduct other interventions to reduce pain, such as such as sucrose intake or breastfeeding [12–15, 17]. Breastfeeding during or shortly before the vaccination has been recommended by the WHO [15]. However, acceptance of breastfeeding is still limited in our institute due to close-minded culture.

This study has some limitations. First, we did not randomize our study groups, because the parents preferred to select the holding positions themselves rather than be randomly assigned to a group; this is a common limitation and risk of bias was present in this study type [35]. At least we were able to determine parental preferences. Second, because the intervention (i.e., the holding position) was performed by the parents, it was nonuniform; however, no unfamiliar holding positions were noted. Third, we did not exclude all possible confounders. For example, bias of measurement may be present although our outcome observer did not know the study purpose. In addition, uneven condition of participants before injection may be a potential confounder. Fourth, the analytic method of this observational study cannot prove causation.

## 5. Conclusions

In this study, we reported the effectiveness of the supine position for reducing acute pain during injection among 2-month-old infants. Holding in the supine position was more effective in relieving vaccination pain than holding in the upright position was. Thus, we recommend that holding in the supine position by parents may aid in reducing vaccination pain in young infants. Nevertheless, positioning on its own did not sufficiently reduce pain. Therefore, additional interventions, including sucrose intake and breastfeeding, in a combination with supine position should be more helpful in reducing pain from injection.

## Figures and Tables

**Table 1:** Infant pain scale.

	Score		
	0	1	2
Crying	No crying	Whimpering	Vigorous crying
Irritability	At rest	Irritation with flexed or extended limbs	Agitation with rigid limbs
Facial expression	Relaxed or smiling	Grimace	Frown with trismus and chin shiver

**Table 2:** Infant demographic data.

Position	Supine	Upright	*p* ^*∗*^
Demographics	*n* = 103	*n* = 179	
Sex			0.374
Male	49 (47.6%)	95 (53.1%)	
Female	54 (52.4%)	84 (46.9%)	
Age (days; means ± SD)	68.4 ± 7.6	67.0 ± 6.8	0.107
Number of vaccines			0.719
Single	19 (18.4%)	30 (16.8%)	
Multiple	84 (81.6%)	149 (83.2%)	
Person holding the infants			0.789
Parents	87 (84.5%)	149 (83.2%)	
Others	16 (15.5%)	30 (16.8%)	
Sucking on a pacifier after injection	2 (1.9%)	1 (0.6%)	0.556

^**∗**^Chi-square test or Student's *t-*test when appropriate.

**Table 3:** Comparison of pain scores between the supine and upright positions (*n* = 282).

Position	Supine	Upright	*p*
Score	*n* = 103	*n* = 179	
Before injection			
Crying	0.11 ± 0.37	0.07 ± 0.28	0.382
Irritability	0.15 ± 0.35	0.09 ± 0.29	0.147
Facial expression	0.12 ± 0.38	0.04 ± 0.21	0.040
Immediately after injection			
Crying	1.13 ± 0.33	1.08 ± 0.27	0.188
Irritability	1.01 ± 0.22	0.94 ± 0.34	0.057
Facial expression	1.07 ± 0.32	0.99 ± 0.28	0.031
30 s after intervention			
Crying	0.53 ± 0.54	0.72 ± 0.48	0.004
Irritability	0.43 ± 0.50	0.58 ± 0.50	0.013
Facial expression	0.46 ± 0.50	0.60 ± 0.49	0.022
180 s after intervention			
Crying	0.08 ± 0.27	0.12 ± 0.35	0.254
Irritability	0.06 ± 0.24	0.09 ± 0.29	0.350
Facial expression	0.07 ± 0.25	0.08 ± 0.28	0.634

**Table 4:** Comparison of pain scales between the supine and upright positions by dichotomized measure (*n* = 282).

Position	Supine	Upright	*p*
Number	*n* = 103	*n* = 179	
Before injection			
Crying	9 (8.7%)	12 (6.7%)	0.531
Irritability	15 (14.6%)	16 (8.9%)	0.146
Facial expression	10 (9.7%)	8 (4.5%)	0.083
Immediately after injection			
Crying	103 (100%)	179 (100%)	—
Irritability	101 (98.1%)	163 (91.1%)	0.021
Facial expression	101 (98.1%)	171 (95.5%)	0.335
30 s after intervention			
Crying	53 (51.5%)	126 (70.4%)	0.001
Irritability	44 (42.7%)	104 (58.1%)	0.013
Facial expression	47 (45.6%)	107 (59.8%)	0.022
180 s after intervention			
Crying	8 (7.8%)	21 (11.7%)	0.291
Irritability	6 (5.8%)	16 (8.9%)	0.348
Facial expression	7 (6.8%)	15 (8.4%)	0.633

**Table 5:** Pain reduction after intervention by a composite grade.

Position	Supine	Upright	*p*
	*n* = 103	*n* = 179	
Pain reduction	*n* (%)	*n* (%)	
30 s after intervention	66 (64.1)	85 (47.5)	0.007
180 s after intervention	98 (95.1)	165 (92.2)	0.339
